# Plasminogen Tochigi mice exhibit phenotypes similar to wild-type mice under experimental thrombotic conditions

**DOI:** 10.1371/journal.pone.0180981

**Published:** 2017-07-07

**Authors:** Yuko Tashima, Fumiaki Banno, Toshiyuki Kita, Yasuyuki Matsuda, Hiroji Yanamoto, Toshiyuki Miyata

**Affiliations:** 1 Department of Molecular Pathogenesis, National Cerebral and Cardiovascular Center, Suita, Osaka, Japan; 2 Laboratory of Neurology and Neurosurgery, National Cerebral and Cardiovascular Center, Suita, Osaka, Japan; 3 Department of Cerebrovascular Medicine, National Cerebral and Cardiovascular Center, Suita, Osaka, Japan; Medical University of South Carolina, UNITED STATES

## Abstract

Plasminogen (Plg) is a precursor of plasmin that degrades fibrin. A race-specific A620T mutation in Plg, also known as Plg-Tochigi, originally identified in a patient with recurrent venous thromboembolism, causes dysplasminogenemia with reduced plasmin activity. The Plg-A620T mutation is present in 3–4% of individuals in East Asian populations, and as many as 50,000 Japanese are estimated to be homozygous for the mutant 620T allele. In the present study, to understand the changes of thrombotic phenotypes in individuals with the mutant 620T allele, we generated knock-in mice carrying the homozygous Plg-A622T mutation (*Plg*^T/T^), an equivalent to the A620T mutation in human Plg. *Plg*^T/T^ mice grew normally but showed severely reduced plasmin activity activated by urokinase, equivalent to ~8% of that in wild-type mice. *In vitro* fibrin clot lysis in plasma was significantly slower in *Plg*^T/T^ mice than in wild-type mice. However, all experimental models of electrolytic deep vein thrombosis, tissue factor-induced pulmonary embolism, transient focal brain ischaemic stroke, or skin-wound healing showed largely similar phenotypes between *Plg*^T/T^ mice and wild-type mice. Protein S-K196E mutation (*Pros1*^E/E^) is a race-specific genetic risk factor for venous thromboembolism. Coexistence in mice of *Plg*^T/T^ and *Pros1*^E/E^ did not affect pulmonary embolism symptoms, compared with those in *Pros1*^E/E^ mice. Hence, the present study showed that the Plg-A622T mutation, which confers ~8% plasmin activity, does not increase the risk of thrombotic diseases in mice under experimental thrombotic conditions and does not modify the thrombotic phenotype observed in *Pros1*^E/E^ mice. *Plg*^T/T^ mice can be used to investigate the potential pathophysiological impact of the Plg-A620T mutation.

## Introduction

Fibrinolysis is an intrinsic system for the decomposition of fibrin clots that is required to maintain vascular patency [1–3]. The primary fibrinolytic enzyme, plasmin, is a serine protease that is converted from plasminogen (Plg) through the cleavage of a single peptide bond by either tissue-type Plg activator (tPA) or urokinase-type Plg activator (uPA).

Plg deficiency has two subtypes, hypoplasminogenemia (type I Plg deficiency) and dysplasminogenemia (type II Plg deficiency). Hypoplasminogenemia is characterized by a reduced Plg activity and antigen level, and the common clinical manifestations among patients with severe hypoplasminogenemia are ligneous conjunctivitis and ligneous gingivitis [4, 5]. Dysplasminogenemia is characterized by reduced Plg activity with normal antigen limits. In 1978, dysplasminogenemia was found in a Japanese patient who had suffered from recurrent venous thromboembolism (VTE) for 15 years [6, 7]. Subsequently, an amino acid substitution of Ala620 to Thr (Plg-A620T, rs121918027, c.1858G>A, Plg-A601T in the mature protein numbering, also known as Plg-Tochigi), close to the active site His622 of the catalytic triad, was identified in patients with dysplasminogenemia [8, 9]. The Plg-A620T mutation, which is absent in white populations, appears with an allele frequency of 0.020 in Japanese [10], 0.015 in Chinese and 0.016 in Koreans [11]. The Japanese population is now approximately 126 million. Extrapolating from these values, we estimate that approximately 1 of every 2,500 Japanese individuals is homozygous for the mutant allele, representing a total of as many as 50,000 individuals.

The relation of the Plg-A620T mutation to thrombotic diseases is debatable. The heterozygous Plg-A620T mutation was originally identified in patients with VTE [6, 9]. Our previous association study of heterozygous Plg-A620T mutation carriers found that this mutation was not a risk factor for VTE [10, 12], but it remains unclear whether the homozygous Plg-A620T mutation confers such a risk due to its rareness. Our recent association study also found that heterozygous Plg-A620T mutation was not a risk factor for atypical hemolytic uremic syndrome [13].

Mouse models are one of the most useful tools to investigate the pathological impact of genetic variants [14]. Even though Plg-knockout mice, a model of hypoplasminogenemia, have been reported to show normal viability, they also express growth retardation, severe spontaneous thrombotic phenotypes, large ischaemic infarction in a brain ischaemia model, and impaired wound healing [1, 15–18]. In addition, Plg-knockout mice showed pseudomembrane diseases such as ligneous conjunctivitis, in which wound healing in fibrin-rich mucous membranes is markedly impaired [19]. Mice with active-site mutated Plg, Plg-S762A, have also been produced and show many of the spontaneous phenotypes observed in Plg-knockout mice [20].

In the present study, to understand the changes of thrombotic phenotypes in humans with the mutant 620T allele, we generated a mouse colony carrying the homozygous Plg-A622T (*Plg*^T/T^) mutation, an equivalent to the human Plg-A620T mutation. We also produced mice carrying both the *Plg*^T/T^ mutation and the protein S-K196E (*Pros1*^E/E^) mutation by crosses. The latter mutation is possessed by 1 in ~55 Japanese, predisposing carriers to thrombosis with an odds ratio of 4.7 for VTE [12]. We have previously generated knock-in mice with *Pros1*^E/E^ and observed the thrombotic phenotypes in *Pros1*^E/E^ mice under the experimental conditions of deep vein thrombosis (DVT) and pulmonary embolism (PE) [21]. We found in the present study that, although mice with the *Plg*^T/T^ mutation showed reduced plasmin activity (~8%) and slower fibrin clot lysis, they showed thrombotic phenotypes largely similar to those of the wild-type mice under experimental thrombotic conditions. Furthermore, we found that mice carrying both the *Plg*^T/T^ mutation and the *Pros1*^E/E^ mutation did not exhibit any aggravation of thrombotic phenotypes compared with mice with the *Pros1*^E/E^ mutation alone.

## Materials and methods

### Ethical statement

All animals were maintained at an animal facility at the National Cerebral and Cardiovascular Center. All animal procedures were approved by the Animal Care and Use Committee of the National Cerebral and Cardiovascular Center (Permit Number: 13004). The animal studies were performed in accordance with institutional and national guidelines and regulations.

### Experimental animals

All mice with the C57BL/6J genetic background were maintained under a regimen of 12h light/12h dark cycles, controlled temperature (22°C), and specific pathogen-free conditions at an animal facility at the National Cerebral and Cardiovascular Center. The mice were fed a normal mouse chow (CLEA Japan, Tokyo, Japan) and tap water ad libitum. The animals were handled by experienced animal takers and veterinarians and were healthy until the experiments were performed. Microbial monitoring was conducted using sentinel animals once every 3 months. Male mice aged 8–12 weeks were used for the phenotypic analyses except for the PE model and the wound-healing model, for which both males and females aged 8–12 week, were used. After the end of the experiment, the mice were directly killed under deep anesthesia.

### Experimental procedures

Mice were anesthetized by intraperitoneal injection of 2,2,2-tribromoethanol or by isoflurane inhalation, and all efforts were made to minimize suffering. The mice for *in vivo* experiments were anesthetized and reflexes were tested to ensure an appropriate level of anesthesia. For an animal survival study using a PE model, the mice were examined for 20 min and then euthanized under deep anesthesia. The mice were constantly watched and monitored throughout the *in vivo* experiments. The duration of the experiments was short and all the mice were euthanized at the time of 20 min. We minimized the observational time to evaluate the PE.

### Generation of Plg A622T knock-in mice

An amino acid-numbering system was adopted with the initial Met residue taken as +1. The targeting vector was constructed in the 5’ and 3’ homology arms by PCR amplification of C57BL/6J genomic DNA as described previously [21–25]. For construction of the 5’ homology arm (6.7 kb), a 5.5-kb *Xho*I-*Hpa*I fragment containing the intron 13-exon 15 of the *Plg* gene and a 1.2-kb *Hpa*I-*Not*I fragment containing the exon 15-intron 16 was amplified and inserted into the *Xho*I-*Not*I sites of the targeting vector. For construction of the 3’ homology arm, a 2.6-kb *Sma*I-*Sac*II fragment containing the introns 16–17 was amplified and cloned into the vector. In exon 15 of the vector, the c.1864G>A (p.A622T) mutation and three translationally silent mutations (c.1857T>G, c.1858C>T, c.1860G>A) creating a new *Hpa*I site were introduced. The vector contains a *lox*P-flanked neomycin resistance cassette (*NEO*) for positive selection and a diphtheria toxin A fragment expression cassette (*DT-A*) for negative selection. The accuracy of the final vector was verified by DNA sequencing. The linearized vector was introduced into C57BL/6J-derived embryonic stem (ES) cells by electroporation. Cells were selected in G418-containing medium and screened by PCR and Southern blot analyses. The targeted ES clones with the normal karyotype were microinjected into BALB/c blastocysts to generate chimeric mice. The resulting male chimeras were bred with wild-type C57BL/6J females (Japan SLC, Hamamatsu, Japan), and F1 offspring with the Plg-A622T mutation were crossed to produce *Alpl*-Cre knock-in mice on the C57BL/6J genetic background (Unitech, Kashiwa, Japan) to excise the *lox*P-flanked *NEO*. The *NEO*-free Plg-A622T mice were re-bred to yield C57BL/6J mice to eliminate the *Alpl*-Cre knock-in allele. The *NEO*-free and Cre-free heterozygous *Plg*^+/T^ mice were interbred to produce homozygous *Plg*^T/T^ mice.

The generation of protein S K196E knock-in (*Pros1*^E/E^) mice on the C57BL/6J genetic background has been described previously [21]. The double homozygous *Plg*^T/T^/*Pros1*^E/E^ mice were obtained by intercrossing of the single-mutant mice.

### Genotypic analysis

Genomic DNA isolated from ES cells or mouse ears was used for genotyping by PCR and Southern blot analyses [21–25]. In PCR analysis, DNA amplification was performed using the primers flanking the *lox*P site, 5’-ACACCTCCGTGTCTCCATTACCTA-3’ and 5’-CTAGCCTCAACCTCACAGAGATCC-3’. The PCR products were analyzed using an Agilent 2100 Bioanalyzer (Agilent Technologies, Santa Clara, CA) to detect the products specific for the wild-type allele (314 bp) and the Plg-A622T allele (415 bp). The primers 5’-GAATGAACGTGCCTTTCTGATTTT-3’ and 5’-CTTCTGGGTCCCTGATGTCACTAC-3’ were used for amplification to verify the precise location of the mutations by DNA sequencing or *Hpa*I-digestion (wild-type: 385 bp; Plg-A622T: 244 and 141 bp). In Southern blot analysis, an alkaline phosphatase-labeled probe was synthesized from a fragment upstream of the 5’-homologous region, hybridized to *Mfe*I/*Hpa*I-digested genomic DNA and detected using a CDP-*Star* detection module (GE Healthcare, Little Chalfont, United Kingdom).

### Quantitative RT-PCR analysis

Total RNA was extracted from the mouse liver using an RNeasy plus mini kit (Qiagen, Hilden, Germany). Quantitative real-time RT-PCR was performed using a QuantiFast Probe RT-PCR kit (Qiagen) with the predesigned primer and probe sets for mouse *Plg* and *Rn18s* (reference gene), according to the manufacturer’s instructions. Amplification and quantification were performed using an Mx 3000P QPCR system (Agilent Technologies). Each RNA sample was analyzed in triplicate. The *Plg* mRNA levels in wild-type mice were defined as 100%.

### Plasma Plg antigens and plasmin activity assays

Blood was collected from anesthetized wild-type and *Plg*^T/T^ mice by cardiac puncture into a syringe primed with a 0.1 volume of 3.8% sodium citrate. Plasma was prepared from blood by centrifugation at 1000 x *g* for 10 min.

Plasma Plg antigens were determined using a mouse Plg total antigen EIA kit (Oxford Biomedical Research, Rochester Hills, MI). In this assay, mouse plasma (100 μl) was incubated with the anti-mouse Plg antibodies coated on a 96-well plate for 30 min. After washing, the plate was incubated with rabbit anti-mouse Plg antibodies for 30 min. Then, the plate was washed again, and incubated with horseradish peroxidase-conjugated anti-rabbit antibodies. After an additional washing the plate was incubated with 3,3',5,5'-tetramethylbenzidine substrate (100 μl). The reaction was stopped after 10 min by adding 1 N H_2_SO_4_ (50 μl). The absorbance of the solution at 450 nm was measured using a Wallac Arvo Sx 1420 Multilabel Counter (Perkin Elmer, Waltham, MA) and the absorbance at 650 nm was subtracted to remove the background. The mean antigen level calculated from 10 wild-type mouse plasma samples was defined as 1 U/ml.

Plasma plasmin activity was determined after treatment with human uPA [26]. Plasma samples (2 μl; n = 10 for each group) were acidified with 0.1 N HCl (10 μl) and 0.38% sodium citrate (3 μl), and incubated for 1 hour at room temperature to denature plasma proteins and to make Plg more effectively activated by uPA. After neutralization by adding 0.1 N NaOH (10 μl) and 50 mM Tris-HCl pH 7.4–0.15 M NaCl (45 μl), human uPA (15 μl; final conc. 1500 U/mL; Merck Millipore, Darmstadt, Germany) was added and the solution was incubated for 30 min at 37°C. Finally, 5 mM chromogenic substrate for plasmin (50 μl; final conc. 1.85 mM, S-2403; pyroGlu-Phe-Lys-pNA·HCl; Chromogenix, Llanelli, UK) was added, followed by incubation for 20 min at 37°C (total vol. 135 μl). The reaction was terminated by adding 1 N H_2_SO_4_ (15 μl). The absorbance at 405 nm was measured with a reference wavelength of 650 nm using a Wallac Arvo Sx 1420 Multilabel Counter. The results for each plasma sample were obtained by subtracting the absorbance value of the sample without plasma (plasma-free sample) from that of the respective sample in plasma. The mean activity in 10 wild-type mouse plasma samples was defined as 100% and that in 10 *Plg*^T/T^ mouse plasma samples was expressed as a percentage of the wild-type activity. Plasma samples (0.2 μl) before and after the treatment with uPA were subjected to SDS-PAGE under reduced conditions, and Western blot using a rabbit anti-mouse Plg polyclonal antibody (Assaypro, St. Charles, MO) was performed as described previously [27].

### Plasma clot lysis analysis

Plasma samples were prepared from anesthetized wild-type (n = 6) and *Plg*^T/T^ (n = 6) mice, as mentioned above. Plasma samples (55 μl) pooled from 6 mice were diluted with 600 mM Hepes buffer pH 7.4 (5 μl) and distilled water (90 μl), and incubated with or without human tPA (20 μl; final conc. 7.5 nM; Merck Millipore) in 20 mM Hepes buffer (pH 7.4) for 30 min at room temperature [28]. Clotting was initiated by adding a mixture (30 μl) of human thrombin (final conc. 14.5 nM; Sigma-Aldrich, St. Louis, MO) and CaCl_2_ (final conc. 20 mM). Turbidities of reaction mixtures (final 200 μL) were monitored every 5 min for 5 hours at the absorbance of 405 nm using a Wallac Arvo Sx 1420 Multilabel Counter. For treatment with a plasmin inhibitor, human α_2_-plasmin inhibitor (final conc. 375 nM; Merck Millipore) was added 5 min before the initiation of clotting.

### DVT model

A DVT model of electrolytic inferior vena cava (IVC) injury established by Diaz *et al*. [29] was slightly modified as described previously [21, 30]. Briefly, wild-type (n = 19) and *Plg*^T/T^ (n = 21) mice were anonymously anesthetized with 2.5% 2,2,2-tribromoethanol and kept at around 37°C. The IVC was exposed, and all side branches between the renal and iliac veins were ligated with a 7–0 polypropylene suture. A 27G stainless-steel needle (NE-115B; Nihon Koden, Tokyo, Japan) was inserted into the IVC (anode), and another needle was inserted subcutaneously (cathode). Using an electric stimulator (SEN-3041; Nihon Koden) with an isolator unit (SS-203J; Nihon Koden), a direct current of 250 μA was applied for 15 min. The needle was gently removed from the IVC and the abdomen was closed by polyglycolic acid suture and cyanoacrylate glue. Thrombus weights were examined in wild-type (n = 9) and *Plg*^T/T^ (n = 9) mice at day 2 after surgery and in wild-type (n = 5) and *Plg*^T/T^ (n = 5) mice at day 7 after surgery. Eight mice died and 4 were excluded due to haemorrhage.

### PE model

A tissue factor-induced PE model was established as described previously [21]. Briefly, wild-type (n = 28), *Plg*^T/T^ (n = 29), *Pros1*^E/E^ (n = 10), and *Plg*^T/T^ and *Pros1*^E/E^ (*Plg*^T/T^*/Pros1*^E/E^, n = 10) mice were anesthetized with 2,2,2-tribromoethanol, and a recombinant human tissue factor reagent containing phospholipids and calcium (Dade Innovin; Siemens AG, Munich, Germany) was infused via the IVC (15 μL/g body weight). Survival time was recorded until 20 min after the infusion, while death was defined as respiratory arrest that persisted for at least 2 min. Two minutes after the respiratory arrest or at the completion of the 20-min observation period, mice were perfused with 0.5 mL of 1% Evans blue via the right ventricle. The lungs were excised and photographed, and the degree of vascular occlusion was evaluated independently by two individuals based on the Evans blue lung perfusion defect scores using a scale of 0 (complete perfusion) to 4 (no perfusion) [21].

### Transient focal brain ischaemia model

Transient focal brain ischaemia was induced using the three-vessel occlusion technique as described previously [21, 24, 25, 31, 32]. Briefly, wild-type (n = 10) and *Plg*^T/T^ (n = 10) mice were anonymously anesthetized by inhalation of isoflurane and kept at around 37°C. The distal M1 portion of the left middle cerebral artery, peripheral to the perforating arteries of the basal ganglia, was permanently occluded by electrocauterization. The bilateral common carotid arteries were transiently occluded for 15 min using vascular clips. After 24 hours, the brains were excised and stained with 2, 3, 5-triphenyl tetrazolium chloride. The infarcted and the total hemispheric areas were measured using WinROOF software (Mitani, Tokyo, Japan). The infarct volume was adjusted for edema by dividing the volume by the edema index (left hemisphere volume / right hemisphere volume). No fatal cases were observed in the transient focal brain ischaemia model.

### Skin-wound healing model

The skin-wound healing model followed the method of Krampert *et al*. [33] that was modified from an original method applied to Plg-knockout mice [17]. Wild-type (n = 11) and *Plg*^T/T^ (n = 11) mice were anesthetized with 2,2,2-tribromoethanol. After shaving and cleaning the dorsal skin, circular full-thickness wounds of 5-mm diameter were made on either side of the midline by excising the skin with a disposable biopsy punch (Kai Industries, Seki, Japan). The same procedure was repeated, generating a total of four wounds in each mouse. The wounds were photographed every other day for two weeks. The wound areas were measured using MetaMorph software (Molecular Devices, Sunnyvale, CA). Changes in the wound area were expressed as a percentage of the original wound area. A wild-type mouse was dead on day 8, and two *Plg*^T/T^ mice were dead on day 4 and day 8, respectively.

### Statistical analysis

Statistical significance was estimated by Student’s *t*-test. Survival rates in the tissue factor-induced PE model were analysed as follows. For each subject, the subject-minutes of follow-up were calculated from the start of the experiment until the subject had survived for 20 min or died, whichever occurred first. Hazard ratios and their 95% confidence intervals were calculated by using the Cox proportional hazard model. Lung perfusion defect scores were assessed by the Mann-Whitney test. The time course of the wound area in the skin-wound healing model was analysed by two-way repeated measures analysis of variance. Differences were considered to be significant at *p* < 0.05. Data are expressed as the means ± standard deviations (SDs).

## Results

### Plasmin activity and Plg antigen levels in *Plg*^T/T^ mice

**Fig 1:**
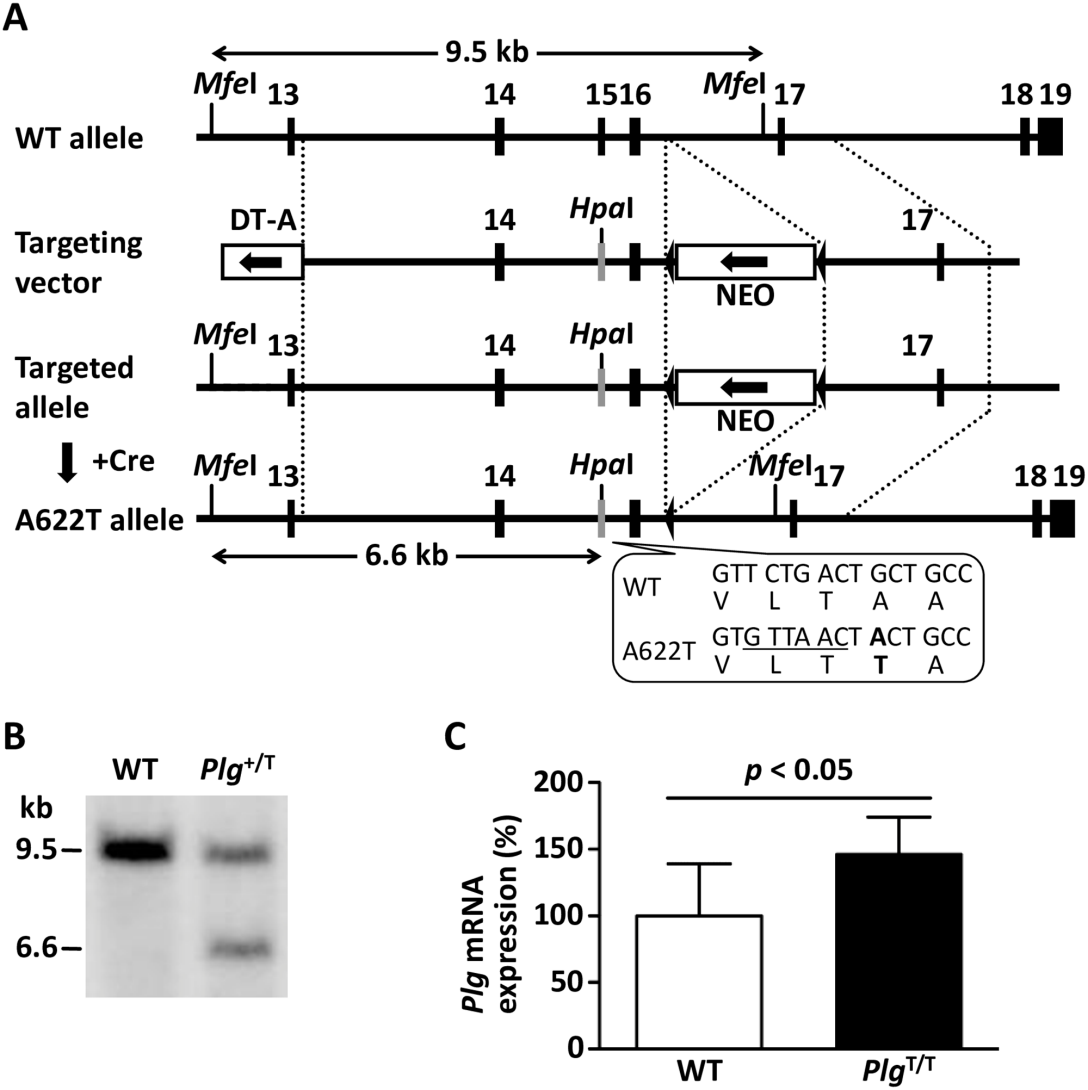
Generation of Plg-A622T mice. (***A***) Structure of the targeted locus in the mouse *Plg* gene. Exons are represented by *filled boxes*. A *lox*P-flanked (*filled triangles*) neomycin-resistance cassette (*NEO*) and a diphtheria toxin A fragment expression cassette (*DT-A*) are indicated by *open boxes* with *arrows* that represent the transcriptional orientation. The A622T mutant allele was produced by homologous recombination and *NEO* deletion mediated by Cre recombinase. The c.1864G>A (p.A622T) mutation and three translationally silent mutations (c.1857T>G, c.1858C>T, c.1860G>A) creating a new *Hpa*I site (*GTTAAC*) were introduced into exon 15. Homologous fragments are indicated by *dotted lines*, while the *Mfe*I-*Hpa*I fragments detected by Southern blot analysis of the wild-type (WT) and Plg-A622T alleles are indicated by *double-headed arrows*. (***B***) Southern blot analysis. Genomic DNA from targeted ES cells was digested with *Mfe*I/*Hpa*I and detected with the specific probe (WT allele: 9.5 kb; Plg-A622T allele: 6.6 kb). (***C***) Quantitative RT-PCR analysis. Total RNA was extracted from mouse liver and subjected to real-time RT-PCR with dual-labeled probes for mouse *Plg* and *Rn18s*. Expression levels of *Plg* mRNA were normalized to *Rn18s* mRNA. Data are the means ± SDs of WT (n = 8) and *Plg*^T/T^ (n = 7) mice. The levels measured in WT mice were defined as 100%.

**Fig 2:**
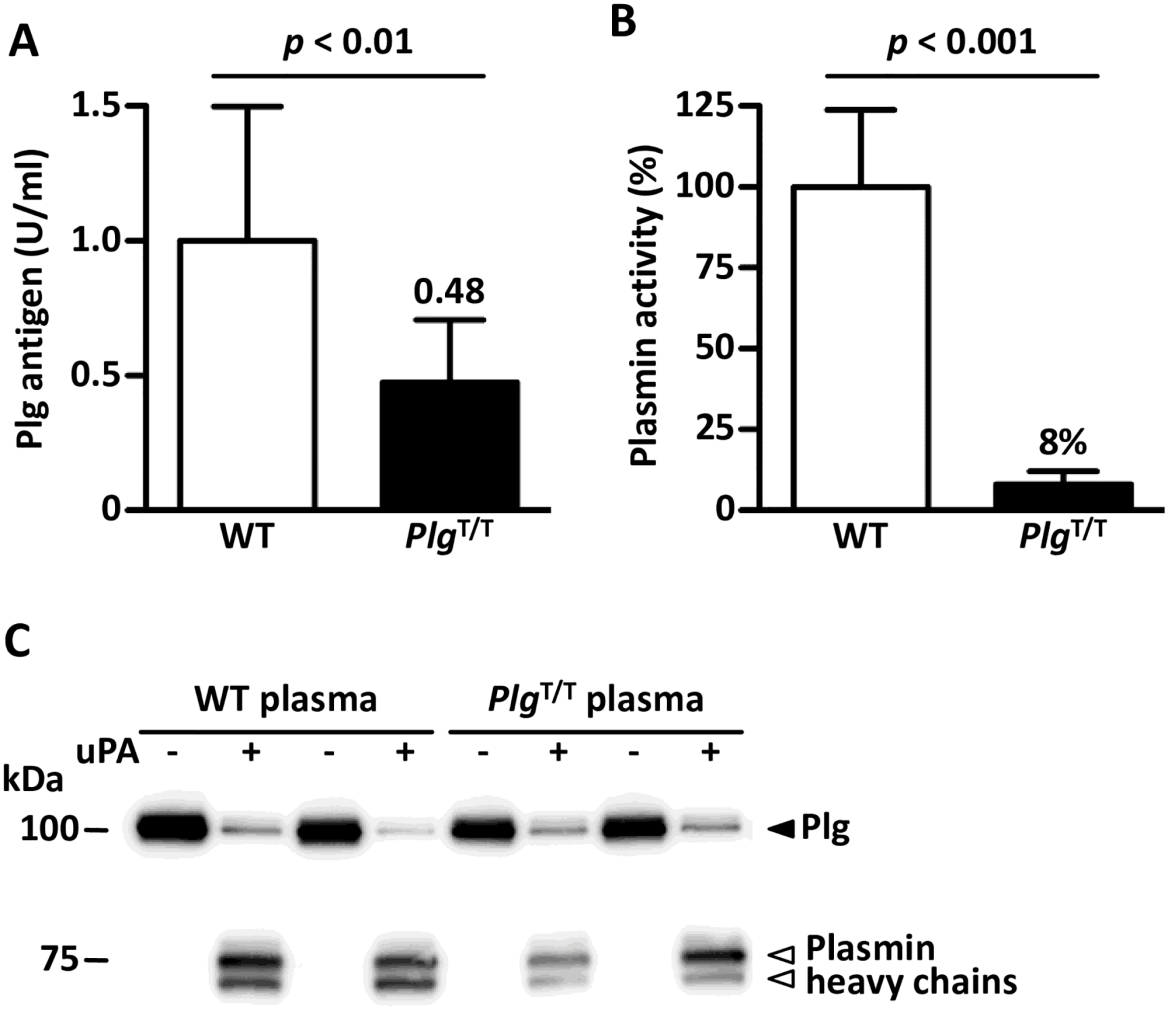
Plasma Plg antigen levels and plasmin activities of wild-type and *Plg*^T/T^ mice. (***A***) Plg antigen levels. Data are the means ± SDs of wild-type (WT, n = 10) and *Plg*^T/T^ (n = 10) mice. The levels measured in WT mice were defined as 1 U/ml. (***B***) Plasmin activities. Plasma from the indicated mice was preincubated with human uPA and reacted with a synthetic substrate, S-2403, for plasmin. Data are the means ± SDs of 10 mice for each genotype. The mean activity measured in WT mice was defined as 100%. (***C***) Western blot analysis of uPA-treated mouse plasma. Two mouse plasma samples of each genotype were incubated with human uPA and separated by SDS-PAGE under reducing conditions. Plg (*black triangle*) and heavy chains of plasmin (*white triangles*) were detected with anti-mouse Plg antibodies.

We generated *Plg*^T/T^ mice on a C57BL/6J genetic background by homologous recombination (Fig 1A) and confirmed the structure of the targeted locus (Fig 1B). The amount of *Plg* mRNA in *Plg*^T/T^ mice (n = 7) was higher than that in wild-type mice (n = 8) (Fig 1C). *Plg*^T/T^ mice were viable and fertile with no signs of Plg-knockout diseases such as ligneous conjunctivitis. The plasma Plg antigen level in *Plg*^T/T^ mice was reduced to approximately half (n = 10, 0.48 ± 0.23 U/ml) of that in wild-type mice (n = 10, 1.00 ± 0.50 U/ml) (Fig 2A), and the plasma plasmin activity after activation with uPA was severely reduced in *Plg*^T/T^ mice (n = 10, 8.1 ± 4.1%) compared to wild-type mice (n = 10, 100 ± 23.8%) (Fig 2B), though the Plg-A622T protein was converted to plasmin by the uPA treatment, as in wild-type Plg (Fig 2C).

**Fig 3:**
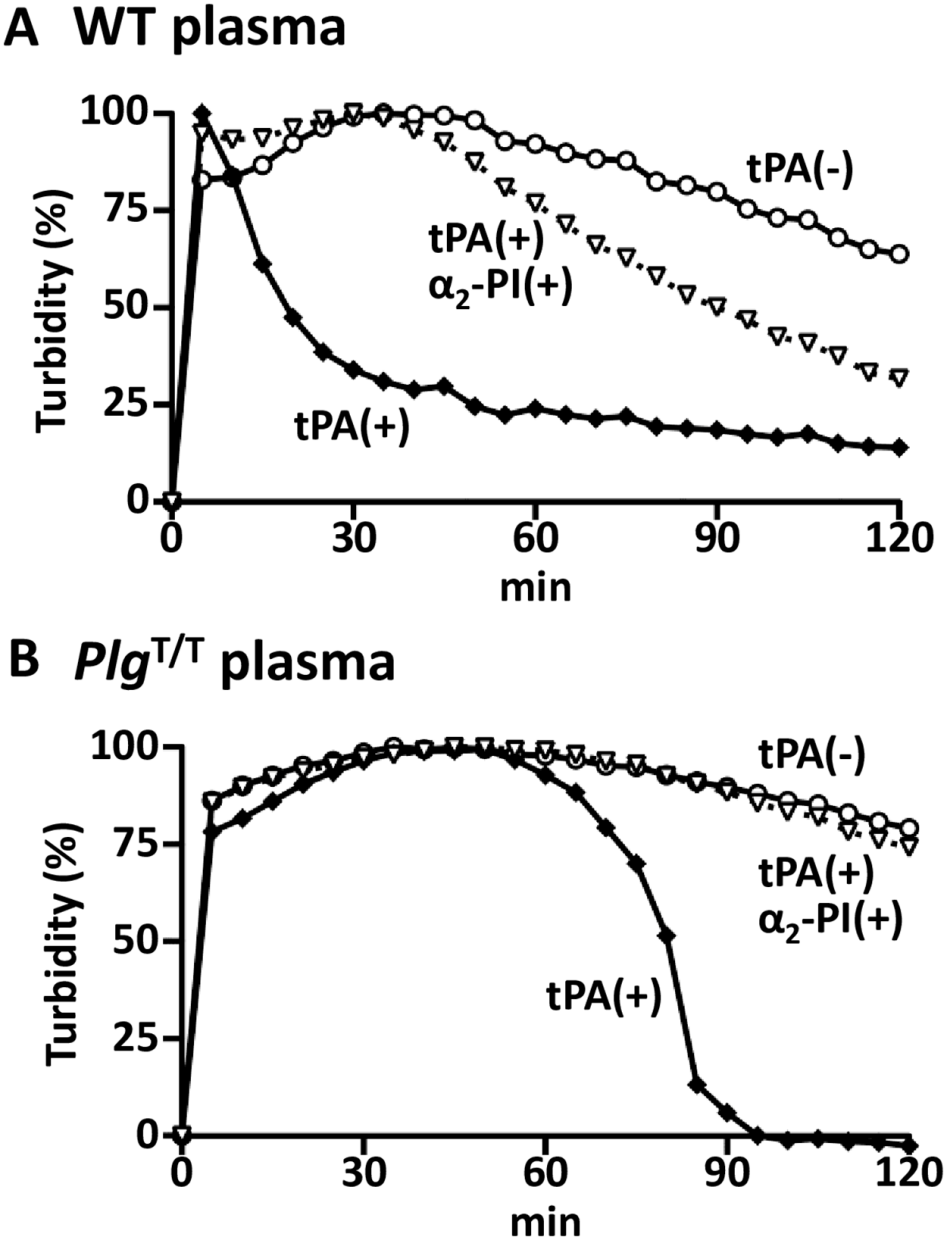
Effect of Plg-A622T mutation on fibrinolysis in plasma. Pooled plasma from 6 wild-type (***A***) or 6 *Plg*^T/T^ (***B***) mice was preincubated in the absence (○) or presence (◆) of human tPA, and clotting was induced with thrombin and CaCl_2_. Human α_2_-plasmin inhibitor (α_2_-PI) was added before the induction of clotting (▽). The turbidity monitored by the absorbance at 405 nm was measured every 5 min as an index of fibrin formation and lysis. The time course of the turbidity was plotted to the maximum absorbance of each sample, which was taken as 100%.

To further characterize the effect of the *Plg*^T/T^ mutation on fibrinolysis, we generated the time course of fibrin clot formation and tPA-mediated clot lysis in plasma samples from wild-type and *Plg*^T/T^ mice (Fig 3). In the presence of tPA, the turbidity of wild-type plasma reached the maximum at 5 min after the addition of a mixture of thrombin and calcium and was declined to half-maximum at around 20 min (Fig 3A), whereas the turbidity of *Plg*^T/T^ plasma gradually reached the maximum at around 45 min and declined to half-maximum at around 80 min (Fig 3B). These profiles indicated that the clot lysis activity in *Plg*^T/T^ plasma was lower than in wild-type plasma. Clots were poorly lysed in the absence of tPA or in the presence of α_2_-plasmin inhibitor in both samples.

### Effects of *Plg*^T/T^ mutation on VTE in DVT and PE models

**Fig 4:**
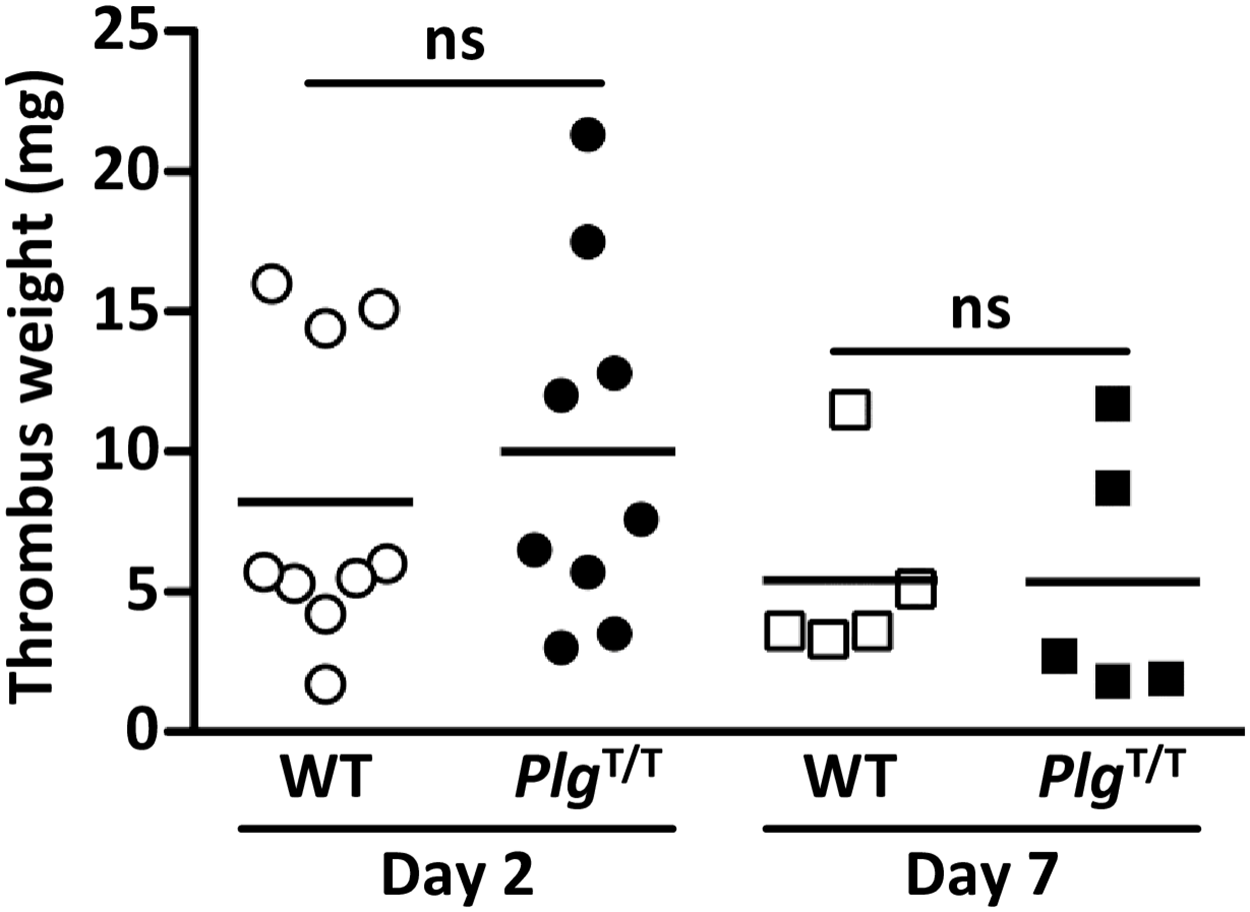
Thrombus weights in electrolytic IVC injury-induced DVT in wild-type and *Plg*^T/T^ mice. On day 2 or day 7 post-injury, thrombi formed in the IVC were weighed. No significant differences (*p* > 0.05) were observed in the thrombus weight between wild-type (WT) and *Plg*^T/T^ mice (n = 9 and n = 9 on day 2, and n = 5 and n = 5 on day 7, respectively). *Circles and squares* represent individual mouse data. *Bars* represent the mean values of groups.

**Fig 5:**
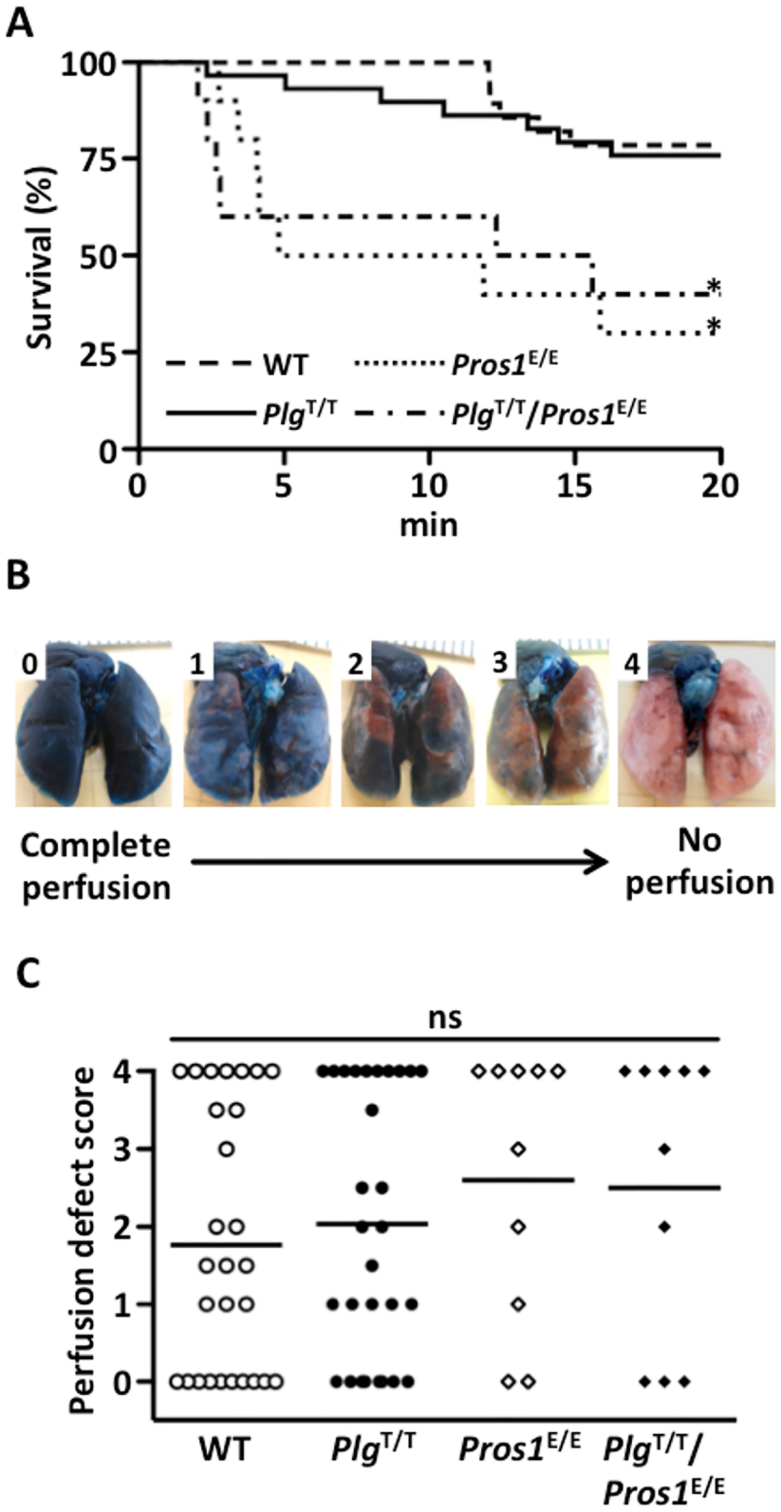
Survival time and lung perfusion defect after tissue factor-induced PE. (***A***) After the tissue factor infusion via IVC, the survival time was recorded until 20 min. No significant difference was observed in the survival between wild-type (WT) (n = 28) and *Plg*^T/T^ (n = 29) mice (hazard ratio, 1.18; 95% confidence intervals, 0.40–3.50), or between *Pros1*^E/E^ (n = 10) and *Plg*^T/T^*/Pros1*^E/E^ (n = 10) mice (hazard ratio, 0.81; 95% confidence intervals, 0.27–2.41). The survival rates of both WT and *Plg*^T/T^ mice were significantly longer than those of *Pros1*^E/E^ and *Plg*^T/T^*/Pros1*^E/E^ mice (WT and *Pros1*^E/E^ mice: hazard ratio, 5.43; 95% confidence intervals, 1.81–16.28; WT and *Plg*^T/T^*/Pros1*^E/E^ mice: hazard ratio, 4.38; 95% confidence intervals, 1.41–13.60; *Plg*^T/T^ and *Pros1*^E/E^ mice: hazard ratio, 4.62; 95% confidence intervals, 1.61–13.28; *Plg*^T/T^ and *Plg*^T/T^*/Pros1*^E/E^ mice: hazard ratio, 3.73; 95% confidence intervals, 1.25–11.11). *Significant difference in comparison to WT mice and *Plg*^T/T^ mice. (***B***) The scale used to measure lung perfusion defect scores. A score of 0 indicates complete perfusion of Evans blue with no occlusion and a score of 4 indicates no Evans blue perfusion with complete occlusion. (***C***) Perfusion defect scores. No significant (ns) differences (*p* > 0.05) were observed among WT (n = 28), *Plg*^T/T^ (n = 29), *Pros1*^E/E^ (n = 10) and *Plg*^T/T^*/Pros1*^E/E^ mice (n = 10). Perfusion defect scores were assessed by the Mann-Whitney test. *Circles* and *diamonds* represent individual mouse data. *Bars* represent the mean values of groups.

To investigate the effects of the *Plg*^T/T^ mutation on VTE, we performed experiments using the DVT and PE models. In the DVT model, we applied electrolytic stimulation to the endothelium of the IVC and evaluated thrombus formation and resolution under continuous blood flow [29]. In both the acute (day 2) and chronic (day 7) phases, the thrombus weight in IVC did not differ between wild-type and *Plg*^T/T^ mice (n = 9 and n = 9 on day 2, and n = 5 and n = 5 on day 7, respectively) (Fig 4). In the PE model, we infused a human tissue factor reagent including phospholipids and calcium into the IVC, and then evaluated 20-min survival (Fig 5A). No significant difference was found in 20-min survival between wild-type and *Plg*^T/T^ mice (n = 28 and n = 29, respectively; hazard ratio, 1.18; 95% confidence intervals, 0.40–3.50). We also evaluated the degree of lung vascular occlusion by perfusion defect scores using Evans blue (Fig 5B). No significant difference was found in perfusion defect scores between wild-type and *Plg*^T/T^ mice (n = 28 and n = 29, respectively) (Fig 5C). These results demonstrate that the *Plg*^T/T^ mutation in mice has no impact on the severity of VTE.

Next, we investigated the possible synergistic effect for disease phenotypes caused by the *Plg*^T/T^ mutation by producing mice carrying both *Plg*^T/T^ and the homozygous protein S-K196E mutation (*Plg*^T/T^*/Pros1*^E/E^). Protein S is an anticoagulant cofactor protein for activated protein C, and the K196E mutation found specifically in the Japanese population predisposes both humans [10, 12] and mice [21] to VTE. Indeed, survival after the induction of PE was significantly reduced in *Pros1*^E/E^ mice compared to wild-type mice (Fig 5A; hazard ratio, 5.43; 95% confidence intervals, 1.81–16.28). However, the survival rates in *Pros1*^E/E^ and *Plg*^T/T^*/Pros1*^E/E^ mice were indistinguishable (Fig 5A; hazard ratio, 0.81; 95% confidence intervals, 0.27–2.41). Therefore, the *Plg*^T/T^ mutation does not aggravate VTE in mice predisposed to thrombosis. No significant difference was found in perfusion defect scores between *Pros1*^E/E^ and *Plg*^T/T^*/Pros1*^E/E^ mice (Fig 5C).

### No exacerbation of *Plg*^T/T^ mutation in the transient focal brain ischaemia model

**Fig 6:**
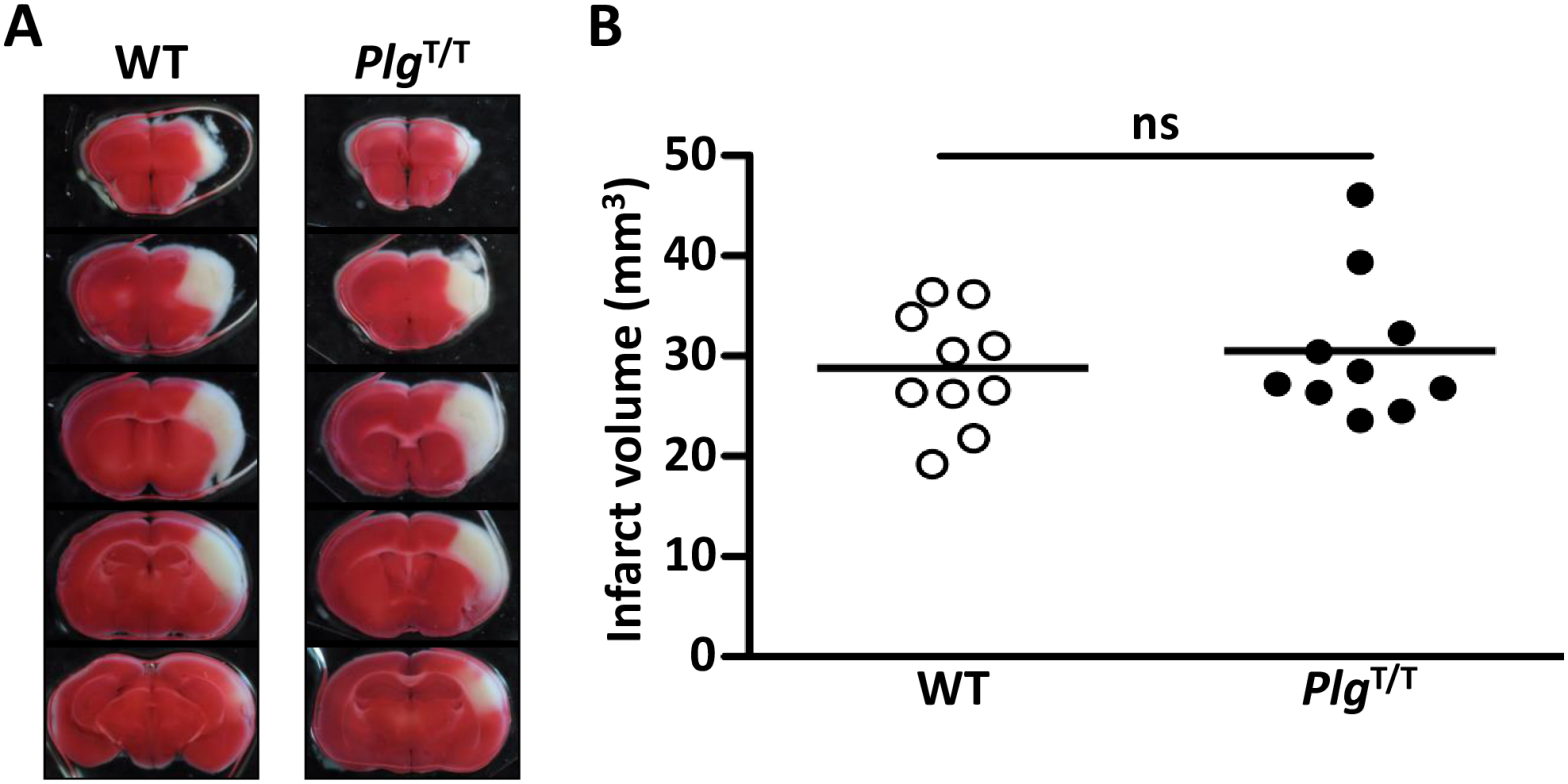
No exacerbation of *Plg*^T/T^ mutation in the transient focal brain ischaemia model. (***A***) Representative images of coronal sections of wild-type (WT) and *Plg*^T/T^ mouse brains. Permanent occlusion of the distal M1 portion of the left middle cerebral artery and 15-min transient occlusion of the bilateral common carotid arteries were applied. After 24 hours, the brains were excised and stained with 2, 3, 5-triphenyl tetrazolium chloride. *White* areas represent brain infarction. (***B***) Infarct volumes. The infarct volume was adjusted for edema by dividing the volume by the edema index (left hemisphere volume / right hemisphere volume). No significant differences (*p* > 0.05) were observed between groups. *Circles* represent individual mouse data. *Bars* represent the mean values of groups.

To examine the effects of *Plg*^T/T^ mutation on arterial occlusive diseases, transient focal brain ischaemia was applied using the three-vessel occlusion technique [21, 24, 25, 31, 32]. This technique avoids the use of any intraluminal foreign materials that may modify the outcome of ischaemic brain injuries by activating coagulation during ischaemia, and produces focal ischaemia-reperfusion for constant cortical infarction [32]. Although early studies suggested an association between the Plg-A620T mutation and ischaemic stroke in young adults [34], we found no difference in brain infarct volumes (adjusted by edema) after ischaemia between wild-type and *Plg*^T/T^ mice (Fig 6). Likewise, the edema index for the evaluation of brain edema in the acute phase did not differ between groups (the edema index: wild-type, 1.07 ± 0.07 (means ± SDs), *Plg*^T/T^, 1.07 ± 0.02 (means ± SDs), *p* = 0.50 by *t*-test). These results indicated that the *Plg*^T/T^ mutation in mice does not expand cerebral ischaemic damage.

### Wound-healing ability in wild-type and *Plg*^T/T^ mice

**Fig 7:**
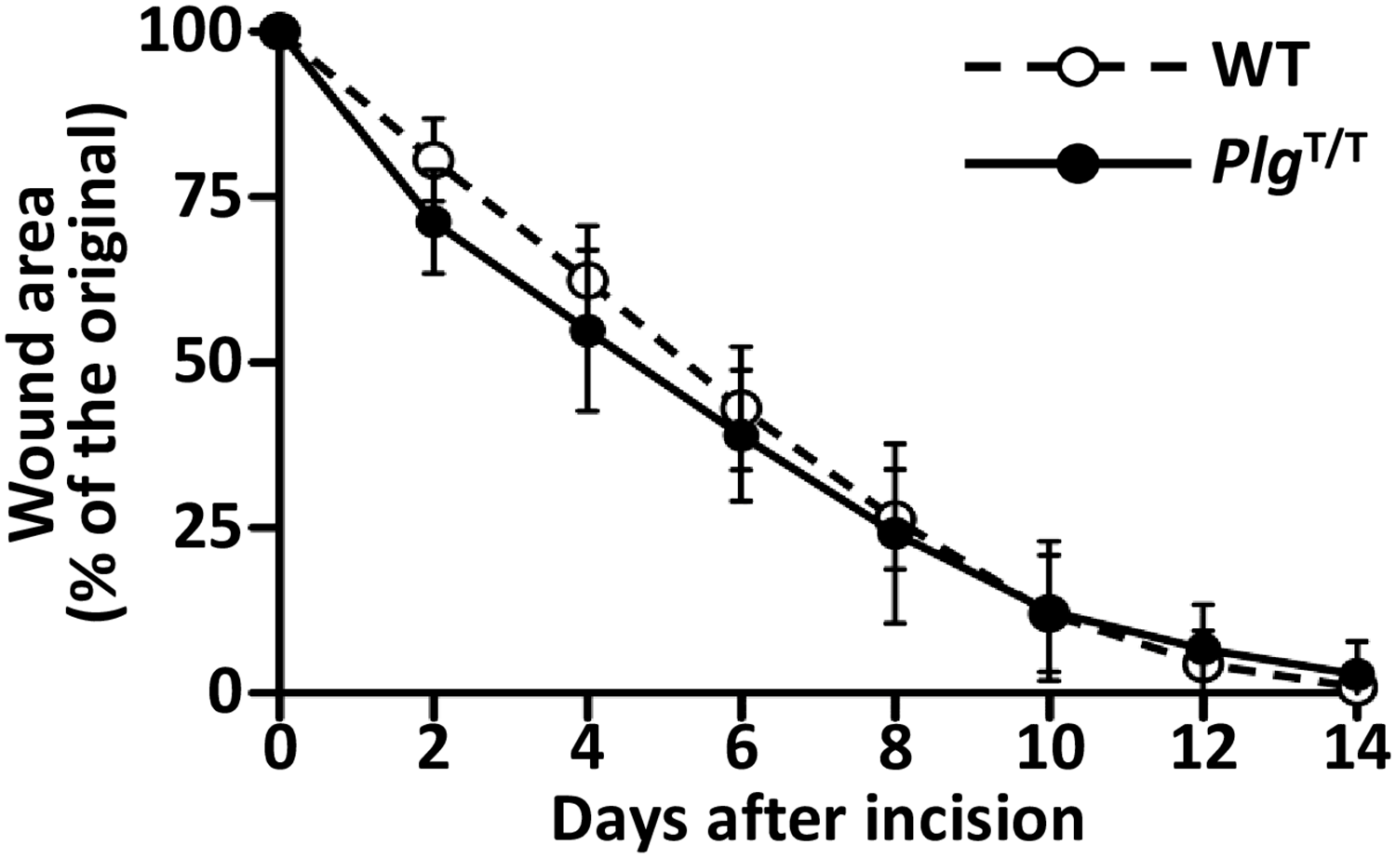
No effects of *Plg*^T/T^ mutation in the skin-wound healing model. The time course of the wound area was measured every other day for two weeks. Data are the means ± SDs of wild-type (WT, n = 10) and *Plg*^T/T^ (n = 9) mice. No significant differences (*p* > 0.05) were observed between groups.

Homozygous Plg-knockout mice are reported to show delayed wound healing [1, 17]. Thus, we investigated how the *Plg*^T/T^ mutation in mice affects the skin-wound healing process. The areas of dorsal skin wounds in wild-type and *Plg*^T/T^ mice were similarly reduced; the wounds in both groups were almost completely closed at two weeks (Fig 7). These results suggest that the low but detectable plasmin activity in *Plg*^T/T^ mice is sufficient to allow for normal wound healing.

## Discussion

We generated *Plg*^T/T^ mice with the Plg-A622T mutation, an equivalent to the race-specific Plg-A620T mutation in humans. Previous studies have shown that Plg-knockout mice developed multiple spontaneous thrombotic lesions in many tissues and exhibited a high level of ischaemic infarction when subjected to brain ischaemia injury [15, 16, 18]. These mice have also been shown to develop pseudomembrane diseases such as ligneous conjunctivitis [19]. In contrast, the present study showed that *Plg*^T/T^ mice were viable and fertile with no signs of spontaneous thrombotic lesions and did not show ligneous conjunctivitis. Even after thrombotic challenges for DVT, PE, or ischaemic stroke, *Plg*^T/T^ mice showed thrombotic phenotypes largely similar to those of the wild-type mice.

To examine the possible synergistic effect caused by the *Plg*^T/T^ mutation, the double homozygous *Plg*^T/T^ and *Pros1*^E/E^ (*Plg*^T/T^*/Pros1*^E/E^) mice were examined to determine whether they showed higher levels of thrombotic characteristics than *Pros1*^E/E^ mice. Our study suggested that mice carrying both the *Plg*^T/T^*/Pros1*^E/E^ mutations showed no aggravation of PE phenotypes compared with mice carrying the *Pros1*^E/E^ mutation alone, suggesting that the Plg-A622T mutation would not be a thrombotic modifier in mice, at least in the case of the tissue factor-induced PE. We speculate that, even though *Plg*^T/T^ mouse plasma showed slower fibrin clot lysis *in vitro*, the reduced but still significant plasmin activity (~8% plasmin activity) would have contributed to the largely similar PE phenotype between the wild-type and *Plg*^T/T^ mice and between the *Pros1*^E/E^ and *Plg*^T/T^*/Pros1*^E/E^ mice. In Plg-knockout mice, fibrinolytic enzymes released from polymorphonuclear leukocytes partly compensated for the deficiency of plasmin-dependent fibrinolysis [35]. Thus, we cannot exclude the possible contribution of leukocyte-derived fibrinolytic enzymes to the dissolution of fibrin clots in *Plg*^T/T^ mice.

In the present study, homozygous *Plg*^T/T^ mice showed severely reduced Plg activity (to ~8% of the wild-type level), with Plg antigen levels approximately half (~48%) those of the wild-type. In humans, a homozygote plasma sample showed severely reduced activity (to ~10% of the control) with a normal antigen level [6]. Therefore, both humans and mice with homozygous mutation show low but significant Plg activity, but their plasma Plg antigen levels differ, with the values being low in mice and normal in humans.

Because *Plg* mRNA in *Plg*^T/T^ mice was increased compared to the level in wild-type mice, the secretion efficiency of the mouse Plg-A622T mutant may have been partially impaired. It is known that the secretion efficiency of the proteins can be affected by subtle structural differences such as missense mutations. Secretory proteins such as Plg have to be correctly folded in the endoplasmic reticulum. In the case that the proteins are not properly folded in the endoplasmic reticulum, the misfolded proteins will not be secreted but degraded [36]. Human and mouse Plg molecules show 78% amino acid identity. This means that 22% of the amino acids are non-conserved, and these non-conserved amino acids could give rise to subtle structural differences between the two molecules. Probably, the mouse Plg mutant with Thr622 would be less properly folded, making it more susceptible to degradation in the endoplasmic reticulum than the human Plg mutant.

Plg-knockout mice and mice with the active site mutation Plg-S762A have previously been produced and characterized [15, 16, 20]. Here, we established a new knock-in mouse line with the *Plg*^T/T^ mutation. The two previous Plg-modified mouse lines held 50% Plg activity in heterozygotes or completely lost Plg activity in homozygotes. On the other hand, homozygous *Plg*^T/T^ mice showed markedly reduced but significant Plg activity (~8%), indicating a unique phenotype compared with the two previous Plg-modified mouse lines. *In vivo* studies using Plg-knockout mice have uncovered many physiological/pathophysiological functions of Plg [1, 37–39]. Hence, the *Plg*^T/T^ mouse line will be useful in future studies to address the unrecognized phenotypes of the *Plg*^T/T^ mutation in mice.

The present study has several limitations. Since heterozygous Plg-A620T mutation was originally identified in patients with VTE [6, 7, 9], we aimed to evaluate the thrombotic risk of this mutation *in vivo*. Therefore, in the present study, we focused on the effects of the Plg-A622T mutation on thrombogenesis and tissue repair in *Plg*^T/T^ mice, and did not examine the effects of this mutation on leukocyte migration, macrophage phagocytosis, or liver injury, all of which have been intensively studied using Plg-knockout mice [37–39]. In addition, we did not examine fibrinolysis models in which the thrombus is treated with exogenous thrombolytics, such as the batroxobin model or the tPA model [40, 41]. A recent study using the thromboembolic stroke model indicated that plasma Plg levels in mice affected dissolution of the middle cerebral artery thrombus [42]. It would be helpful to examine these experimental models in the future. A limitation of the present study includes the lack of validation that the models used are sensitive to Plg.

Another limitation is that the thrombotic models of DVT, PE and stroke used in the present study constitute “artificial” challenges and may not fully reflect the thrombotic conditions occurring in humans. Humans carry a large variety of genetic variations and encounter a wide range of environmental stimuli, some of which may increase thrombotic risk. Therefore, it should be noted that our results do not exclude the possibility that the Plg-A620T mutation in humans plays a role in thrombosis that could be manifested under conditions other than those used here.

In summary, we developed an original colony of *Plg*^T/T^ mice to investigate the thrombotic risk of homozygous *Plg*^T/T^ mutation. Our data suggest that mice with severely reduced Plg activity caused by the homozygous Plg-A622T mutation showed phenotypes similar to wild-type mice under the experimental conditions of VTE, PE, ischaemic stroke and wound healing, and did not exhibit aggravation of thrombosis even in the presence of a protein S mutation conferring predisposition to thrombosis.

## References

[1] Castellino FJ, Ploplis VA. Structure and function of the plasminogen/plasmin system. Thromb Haemost. 2005;93(4):647–54. doi: 10.1160/TH04-12-0842 . 15841308 10.1160/TH04-12-0842

[2] Schuster V, Hugle B, Tefs K. Plasminogen deficiency. J Thromb Haemost. 2007;5(12):2315–22. doi: 10.1111/j.1538-7836.2007.02776.x . 17900274 10.1111/j.1538-7836.2007.02776.x

[3] Rijken DC, Lijnen HR. New insights into the molecular mechanisms of the fibrinolytic system. J Thromb Haemost. 2009;7(1):4–13. doi: 10.1111/j.1538-7836.2008.03220.x . 19017261 10.1111/j.1538-7836.2008.03220.x

[4] Schuster V, Mingers AM, Seidenspinner S, Nussgens Z, Pukrop T, Kreth HW. Homozygous mutations in the plasminogen gene of two unrelated girls with ligneous conjunctivitis. Blood. 1997;90(3):958–66. . 9242524

[5] Tefs K, Gueorguieva M, Klammt J, Allen CM, Aktas D, Anlar FY, et al Molecular and clinical spectrum of type I plasminogen deficiency: A series of 50 patients. Blood. 2006;108(9):3021–6. doi: 10.1182/blood-2006-04-017350 . 16849641 10.1182/blood-2006-04-017350

[6] Aoki N, Moroi M, Sakata Y, Yoshida N, Matsuda M. Abnormal plasminogen. A hereditary molecular abnormality found in a patient with recurrent thrombosis. J Clin Invest. 1978;61(5):1186–95. doi: 10.1172/JCI109034 ; 659588 10.1172/JCI109034 PMC372639

[7] Sakata Y, Aoki N. Molecular abnormality of plasminogen. J Biol Chem. 1980;255(11):5442–7. . 6445365

[8] Miyata T, Iwanaga S, Sakata Y, Aoki N. Plasminogen Tochigi: inactive plasmin resulting from replacement of alanine-600 by threonine in the active site. Proc Natl Acad Sci U S A. 1982;79(20):6132–6. ; 6216475 10.1073/pnas.79.20.6132 PMC347073

[9] Ichinose A, Espling ES, Takamatsu J, Saito H, Shinmyozu K, Maruyama I, et al Two types of abnormal genes for plasminogen in families with a predisposition for thrombosis. Proc Natl Acad Sci U S A. 1991;88(1):115–9. ; 1986355 10.1073/pnas.88.1.115 PMC50760

[10] Miyata T, Kimura R, Kokubo Y, Sakata T. Genetic risk factors for deep vein thrombosis among Japanese: importance of protein S K196E mutation. Int J Hematol. 2006;83(3):217–23. Epub 2006/05/25. doi: 10.1532/IJH97.A20514 . 16720551 10.1532/IJH97.A20514

[11] Ooe A, Kida M, Yamazaki T, Park SC, Hamaguchi H, Girolami A, et al Common mutation of plasminogen detected in three Asian populations by an amplification refractory mutation system and rapid automated capillary electrophoresis. Thromb Haemost. 1999;82(4):1342–6. . 10544925

[12] Kimura R, Honda S, Kawasaki T, Tsuji H, Madoiwa S, Sakata Y, et al Protein S-K196E mutation as a genetic risk factor for deep vein thrombosis in Japanese patients. Blood. 2006;107(4):1737–8. Epub 2006/02/08. doi: 10.1182/blood-2005-09-3892 . 16461766 10.1182/blood-2005-09-3892

[13] Miyata T, Uchida Y, Yoshida Y, Kato H, Matsumoto M, Kokame K, et al No association between dysplasminogenemia with p.Ala620Thr mutation and atypical hemolytic uremic syndrome. Int J Hematol. 2016;104:223–7. doi: 10.1007/s12185-016-2021-3 . 27194432 10.1007/s12185-016-2021-3

[14] MacArthur DG, Manolio TA, Dimmock DP, Rehm HL, Shendure J, Abecasis GR, et al Guidelines for investigating causality of sequence variants in human disease. Nature. 2014;508(7497):469–76. doi: 10.1038/nature13127 ; 24759409 10.1038/nature13127 PMC4180223

[15] Bugge TH, Flick MJ, Daugherty CC, Degen JL. Plasminogen deficiency causes severe thrombosis but is compatible with development and reproduction. Genes Dev. 1995;9(7):794–807. . 7705657 10.1101/gad.9.7.794

[16] Ploplis VA, Carmeliet P, Vazirzadeh S, Van Vlaenderen I, Moons L, Plow EF, et al Effects of disruption of the plasminogen gene on thrombosis, growth, and health in mice. Circulation. 1995;92(9):2585–93. . 7586361 10.1161/01.cir.92.9.2585

[17] Romer J, Bugge TH, Pyke C, Lund LR, Flick MJ, Degen JL, et al Impaired wound healing in mice with a disrupted plasminogen gene. Nat Med. 1996;2(3):287–92. . 8612226 10.1038/nm0396-287

[18] Nagai N, De Mol M, Lijnen HR, Carmeliet P, Collen D. Role of plasminogen system components in focal cerebral ischemic infarction: a gene targeting and gene transfer study in mice. Circulation. 1999;99(18):2440–4. . 10318667 10.1161/01.cir.99.18.2440

[19] Drew AF, Kaufman AH, Kombrinck KW, Danton MJ, Daugherty CC, Degen JL, et al Ligneous conjunctivitis in plasminogen-deficient mice. Blood. 1998;91(5):1616–24. . 9473227

[20] Iwaki T, Malinverno C, Smith D, Xu Z, Liang Z, Ploplis VA, et al The generation and characterization of mice expressing a plasmin-inactivating active site mutation. J Thromb Haemost. 2010;8(10):2341–4. doi: 10.1111/j.1538-7836.2010.03995.x . 20653841 10.1111/j.1538-7836.2010.03995.x PMC2965814

[21] Banno F, Kita T, Fernandez JA, Yanamoto H, Tashima Y, Kokame K, et al Exacerbated venous thromboembolism in mice carrying protein S K196E mutation. Blood. 2015;126(19):2247–53. doi: 10.1182/blood-2015-06-653162 . 26251307 10.1182/blood-2015-06-653162 PMC4635119

[22] Okuda T, Higashi Y, Kokame K, Tanaka C, Kondoh H, Miyata T. Ndrg1-deficient mice exhibit a progressive demyelinating disorder of peripheral nerves. Mol Cell Biol. 2004;24(9):3949–56. Epub 2004/04/15. doi: 10.1128/MCB.24.9.3949-3956.2004 ; 15082788 10.1128/MCB.24.9.3949-3956.2004 PMC387740

[23] Banno F, Kokame K, Okuda T, Honda S, Miyata S, Kato H, et al Complete deficiency in ADAMTS13 is prothrombotic, but it alone is not sufficient to cause thrombotic thrombocytopenic purpura. Blood. 2006;107(8):3161–6. Epub 2005/12/22. doi: 10.1182/blood-2005-07-2765 . 16368888 10.1182/blood-2005-07-2765

[24] Yamamoto H, Kokame K, Okuda T, Nakajo Y, Yanamoto H, Miyata T. NDRG4 protein-deficient mice exhibit spatial learning deficits and vulnerabilities to cerebral ischemia. J Biol Chem. 2011;286(29):26158–65. Epub 2011/06/04. doi: 10.1074/jbc.M111.256446 ; 21636852 10.1074/jbc.M111.256446 PMC3138246

[25] Eura Y, Yanamoto H, Arai Y, Okuda T, Miyata T, Kokame K. Derlin-1 deficiency is embryonic lethal, Derlin-3 deficiency appears normal, and Herp deficiency is intolerant to glucose load and ischemia in mice. PLoS One. 2012;7(3):e34298 Epub 2012/04/06. doi: 10.1371/journal.pone.0034298 ; 22479592 10.1371/journal.pone.0034298 PMC3315519

[26] Barrios M, Rodriguez-Acosta A, Gil A, Salazar AM, Taylor P, Sanchez EE, et al Comparative hemostatic parameters in BALB/c, C57BL/6 and C3H/He mice. Thromb Res. 2009;124(3):338–43. doi: 10.1016/j.thromres.2008.11.001 . 19101712 10.1016/j.thromres.2008.11.001

[27] Banno F, Kaminaka K, Soejima K, Kokame K, Miyata T. Identification of strain-specific variants of mouse Adamts13 gene encoding von Willebrand factor-cleaving protease. J Biol Chem. 2004;279(29):30896–903. Epub 2004/05/12. doi: 10.1074/jbc.M314184200 . 15136581 10.1074/jbc.M314184200

[28] Parker AC, Mundada LV, Schmaier AH, Fay WP. Factor V^Leiden^ inhibits fibrinolysis in vivo. Circulation. 2004;110(23):3594–8. doi: 10.1161/01.CIR.0000148781.87906.C0 . 15569834 10.1161/01.CIR.0000148781.87906.C0

[29] Diaz JA, Alvarado CM, Wrobleski SK, Slack DW, Hawley AE, Farris DM, et al The electrolytic inferior vena cava model (EIM) to study thrombogenesis and thrombus resolution with continuous blood flow in the mouse. Thromb Haemost. 2013;109(6):1158–69. doi: 10.1160/TH12-09-0711 . 23571406 10.1160/TH12-09-0711 PMC4822196

[30] Tashima Y, Banno F, Akiyama M, Miyata T. Influence of ADAMTS13 deficiency on venous thrombosis in mice. Thromb Haemost. 2015;114(1):206–7. doi: 10.1160/TH14-08-0656 . 25855507 10.1160/TH14-08-0656

[31] Kita T, Banno F, Yanamoto H, Nakajo Y, Iihara K, Miyata T. Large infarct and high mortality by cerebral ischemia in mice carrying the factor V Leiden mutation. J Thromb Haemost. 2012;10(7):1453–5. Epub 2012/05/15. doi: 10.1111/j.1538-7836.2012.04776.x . 22578082 10.1111/j.1538-7836.2012.04776.x

[32] Yang D, Nakajo Y, Iihara K, Kataoka H, Nakagawara J, Zhao Q, et al An integrated stroke model with a consistent penumbra for the assessment of neuroprotective interventions. Eur Neurol. 2014;71(1–2):4–18. Epub 2014/02/15. doi: 10.1159/000356048 . 24525475 10.1159/000356048

[33] Krampert M, Kuenzle S, Thai SN, Lee N, Iruela-Arispe ML, Werner S. ADAMTS1 proteinase is up-regulated in wounded skin and regulates migration of fibroblasts and endothelial cells. J Biol Chem. 2005;280(25):23844–52. doi: 10.1074/jbc.M412212200 . 15843381 10.1074/jbc.M412212200

[34] Nagayama T, Shinohara Y, Nagayama M, Tsuda M, Yamamura M. Congenitally abnormal plasminogen in juvenile ischemic cerebrovascular disease. Stroke. 1993;24(12):2104–7. . 8248995 10.1161/01.str.24.12.2104

[35] Zeng B, Bruce D, Kril J, Ploplis V, Freedman B, Brieger D. Influence of plasminogen deficiency on the contribution of polymorphonuclear leucocytes to fibrin/ogenolysis: studies in plasminogen knock-out mice. Thromb Haemost. 2002;88(5):805–10. . 12428098

[36] Wang M, Kaufman RJ. Protein misfolding in the endoplasmic reticulum as a conduit to human disease. Nature. 2016;529(7586):326–35. doi: 10.1038/nature17041 . 26791723 10.1038/nature17041

[37] Busuttil SJ, Ploplis VA, Castellino FJ, Tang L, Eaton JW, Plow EF. A central role for plasminogen in the inflammatory response to biomaterials. J Thromb Haemost. 2004;2(10):1798–805. doi: 10.1111/j.1538-7836.2004.00916.x . 15456492 10.1111/j.1538-7836.2004.00916.x

[38] Kawao N, Nagai N, Ishida C, Okada K, Okumoto K, Suzuki Y, et al Plasminogen is essential for granulation tissue formation during the recovery process after liver injury in mice. J Thromb Haemost. 2010;8(7):1555–66. doi: 10.1111/j.1538-7836.2010.03870.x . 20345714 10.1111/j.1538-7836.2010.03870.x

[39] Das R, Ganapathy S, Settle M, Plow EF. Plasminogen promotes macrophage phagocytosis in mice. Blood. 2014;124(5):679–88. doi: 10.1182/blood-2014-01-549659 ; 24876560 10.1182/blood-2014-01-549659 PMC4118484

[40] Wu C, Dong N, da Cunha V, Martin-McNulty B, Tran K, Nagashima M, et al Activated thrombin-activatable fibrinolysis inhibitor attenuates spontaneous fibrinolysis of batroxobin-induced fibrin deposition in rat lungs. Thromb Haemost. 2003;90(3):414–21. doi: 10.1160/TH02-09-0104 . 12958609 10.1160/TH02-09-0104

[41] Vercauteren E, Emmerechts J, Peeters M, Hoylaerts MF, Declerck PJ, Gils A. Evaluation of the profibrinolytic properties of an anti-TAFI monoclonal antibody in a mouse thromboembolism model. Blood. 2011;117(17):4615–22. doi: 10.1182/blood-2010-08-303677 . 21343611 10.1182/blood-2010-08-303677

[42] Singh S, Houng AK, Wang D, Reed GL. Physiologic variations in blood plasminogen levels affect outcomes after acute cerebral thromboembolism in mice: a pathophysiologic role for microvascular thrombosis. J Thromb Haemost. 2016 doi: 10.1111/jth.13390 . 27319402 10.1111/jth.13390 PMC5035596

